# Intraoperative Esketamine and Postpartum Depression Among Women With Cesarean Delivery: A Randomized Clinical Trial

**DOI:** 10.1001/jamanetworkopen.2024.59331

**Published:** 2025-02-13

**Authors:** Li Ren, Ting Zhang, Bingyu Zou, Xin Su, Yi Tao, Jie Yang, Feng Lv, Ping Li, Fangliang Peng, Gangming Wu

**Affiliations:** 1 Department of Anesthesiology, The First Affiliated Hospital of Chongqing Medical University, Chongqing, China; 2 Department of Phase I Clinical Trial Ward, The First Affiliated Hospital of Chongqing Medical University, Chongqing, China; 3 Department of Obstetrics, The First Affiliated Hospital of Chongqing Medical University, Chongqing, China

## Abstract

**Importance:**

Esketamine has been found to reduce the incidence of postpartum depression (PPD) in randomized clinical trials. However, current evidence from randomized clinical trials does not reflect esketamine’s efficacy in clinical settings.

**Objective:**

To assess the clinical efficacy of intraoperative esketamine administration for preventing PPD among women who underwent cesarean delivery.

**Design, Setting, and Participants:**

This randomized clinical trial was conducted at The First Affiliated Hospital of Chongqing Medical University in Chongqing, China, from March 2023 to February 2024. Pregnant patients admitted for cesarean delivery were included, while those with intellectual dysfunction or contraindications to esketamine were excluded. All participants were assigned randomly to either the esketamine group or control group in a 1:1 ratio. Data analysis was based on the intention-to-treat principle.

**Interventions:**

Patients in the esketamine group received an infusion of 0.25 mg/kg esketamine in 20 mL of saline over 20 minutes, whereas patients in the control group received 20 mL saline over 20 minutes.

**Main Outcomes and Measures:**

The primary outcome was the incidence of PPD at 6 weeks post partum. PPD was assessed using the Edinburgh Postnatal Depression Scale.

**Results:**

A total of 308 pregnant women were randomly assigned to 1 of 2 groups: esketamine (n = 154; mean [SD] patient age, 31.57 [4.26] years) and control (n = 154; mean [SD] patient age, 32.53 [7.74] years). Incidence of PPD was significantly lower in the esketamine group compared with the control group at 6 weeks post partum (10.4% [16] vs 19.5% [30]; relative risk, 0.53; 95% CI, 0.30-0.93; *P* = .02).

**Conclusions and Relevance:**

This randomized clinical trial demonstrated esketamine’s advantage in reducing the incidence of PPD at 6 weeks post partum in patients who underwent cesarean delivery. The efficacy and safety of esketamine in preventing PPD warrant further investigation in clinical practice.

**Trial Registration:**

Chinese Clinical Trial Registry Identifier: ChiCTR2200065494

## Key Points

**Question:**

Does intraoperative esketamine infusion prevent postpartum depression (PPD) among women with cesarean delivery in actual clinical practice?

**Findings:**

In this randomized clinical trial involving 308 women who underwent cesarean delivery, intraoperative esketamine infusion significantly reduced the incidence of PPD at 6 weeks post partum.

**Meaning:**

Findings of this trial indicate the efficacy and safety of esketamine in preventing PPD among patients with cesarean delivery and warrant further investigation in clinical practice.

## Teaser

This randomized clinical trial evaluates the effect of esketamine infusion during cesarean delivery on postpartum depression 6 weeks after delivery.

## Introduction

Postpartum depression (PPD) is a common issue globally, affecting approximately 17.7% of women after delivery.^1^ This problem is particularly pronounced in China, with an annual incidence of approximately 21.4%.^2^ PPD adversely affects maternal social functioning, family relationships, and the parent-child bonding.^3,4^ Compared with women who delivered vaginally, those who had a cesarean delivery are more likely to experience PPD.^5,6^ Over the past 2 decades, ketamine or esketamine (dextro-isomer of ketamine) has been recognized as an important breakthrough in depression treatment due to its rapid antidepressant effect.^7^ As a result, ketamine or esketamine is being explored for use in treating PPD.

Multiple high-quality randomized clinical trials (RCTs) have investigated the effect of esketamine on PPD in women who had undergone cesarean delivery; however, their conclusions were inconsistent.^8,9,10,11^ Furthermore, an increasing number of researchers conduct meta-analyses to summarize the role of ketamine or esketamine in PPD, with recent meta-analyses showing beneficial findings.^12,13,14^ Yet such meta-analyses consistently found insufficient evidence to effectively inform clinical decision-making. In our opinion, this lack of evidence is due, in part, to a decrease in effectiveness between the RCT design and clinical setting. The conclusion from RCTs could be overestimating benefits and underestimating harm.^15^ Additionally, clinical practice defined by RCTs differs substantially from actual clinical practice. All of these factors may mask the effectiveness of esketamine for PPD in an actual clinical setting.

In contrast to explanatory trials, such as RCTs, pragmatic clinical trials have recently gained attention because they strive to use conditions that minimize the likelihood of demonstrating the treatment effect. Generally, pragmatic trials are designed to examine the comparative balance of benefits and risks of the intervention at the individual or population level^16^ in a clinical setting and to confirm more generalizable results.^17^ There is a growing viewpoint that assessment of a treatment should begin in a controlled environment using an explanatory trial and progress to the clinical setting through a pragmatic trial.^18^ Pragmatic trials are more practical, inclusive, engaged, and relevant than RCTs. The conclusions drawn from pragmatic trials can more accurately reflect the effect of the intervention in everyday practice. Furthermore, they (ie, pragmatic trials) also can yield evidence that can be customized to patient groups frequently underrepresented in RCTs.^17^ However, to our knowledge, there is a lack of evidence from pragmatic trials regarding the effect of esketamine on PPD in women with cesarean delivery. Therefore, the objective of this pragmatic trial was to assess the clinical efficacy of intraoperative esketamine administration for preventing PPD among women who underwent cesarean delivery. We hypothesized that patients receiving esketamine would exhibit a lower incidence of PPD, as measured by the Edinburgh Postnatal Depression Scale, compared with those without esketamine use.

## Methods

This study was a single-center, double-blind pragmatic clinical trial with a balanced 1:1 randomization (see trial protocol in Supplement 1). It was conducted at The First Affiliated Hospital of Chongqing Medical University in Chongqing, China, between March 2023 and February 2024. The study was approved by the ethics committee of The First Affiliated Hospital of Chongqing Medical University. Eligible patients were informed of the study and provided written informed consent before surgery. We followed the Consolidated Standards of Reporting Trials (CONSORT) reporting guideline.

### Participants

Pregnant women admitted for cesarean deliveries with intraspinal anesthesia were eligible for inclusion. Exclusion criteria were conditions contraindicating esketamine, including (1) severe cardiovascular or cerebrovascular diseases, severe gestational hypertension, and preeclampsia or eclampsia; (2) full stomach or intraocular hypertension; (3) uncontrolled hyperthyroidism; (4) a history of drug use; and (5) intellectual dysfunction.

### Randomization and Blinding

**Figure 1.**
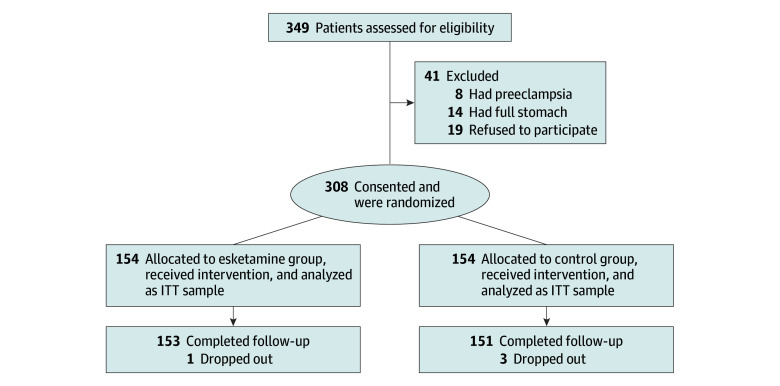
Study Flow Diagram ITT indicates intention to treat.

All eligible patients were assigned randomly to either the esketamine group or control group in a 1:1 ratio using a computer-generated random number sequence (Figure 1). Group allocation information was sealed in opaque envelopes. Before anesthesia administration, the study assistant, who was blinded to the protocol, opened the envelopes according to recruitment sequence and prepared the study drugs for the patients (esketamine group: 0.25 mg/kg esketamine diluted in 20 mL saline with a 20-mL syringe; control group: 20 mL saline with a 20-mL syringe). All patients and outcomes assessors were blinded to the group assignments. The anesthesiologists and health care team members involved in conducting the cesarean delivery were not involved in the follow-up surveys.

### Perioperative Care and Group Assignment

Routine perioperative care was provided to each patient. At hospital admission, patients scheduled for cesarean delivery received routine preoperative preparations and education from physicians. Anesthesiologists conducted preoperative visits the day before elective surgeries or rapid evaluation in the operating room for emergencies. Intraoperative monitoring included electrocardiography, blood pressure reading, and oxygen saturation level, with provision of low-flow oxygen. The puncture site and anesthesia technique (epidural, spinal, or combined spinal-epidural anesthesia) were determined by the anesthesiologist, targeting a sensory block from T6 to T4. After anesthesia was completed, the patient was routinely repositioned to a left lateral tilt of 30° to prevent supine hypotension syndrome. If necessary, vasopressors, such as ephedrine and phenylephrine, or volume therapy were used for hemodynamic stabilization.

Cesarean delivery was performed through a transverse lower uterine segment incision. Forceps were used if deemed necessary by the obstetrician to assist delivery. After fetal delivery, patients in the esketamine group received 0.25 mg/kg esketamine in 20 mL of 0.9% saline over 20 minutes, whereas those in the control group received 20 mL of 0.9% saline over 20 minutes. Oxytocin, ergometrine, or carboprost tromethamine was administered to enhance uterine contractility. Additional surgical interventions to prevent postpartum hemorrhage, such as the B-Lynch suture or uterine balloon tamponade, were performed as needed by the obstetrician. At the end of surgery, patient-controlled intravenous analgesia (PCIA) was initiated for each patient. The formula of PCIA contained fentanyl, 1 mg, and dexamethasone, 10 mg, dissolved in saline to a 100-mL solution.

PCIA was programmed for continuous infusion at 1.5 mL per hour with a bolus dose of 2 mL allowed every 15 minutes. The acute pain service team adjusted the PCIA parameters based on patient pain intensity, with discontinuation planned within 72 hours postoperatively based on patient-reported pain levels. Additionally, the ultrasonography-guided nerve block, such as transversus abdominis plane block or quadratus lumborum block, was performed based on anesthesiologist preference and patient need.

### Primary and Secondary Outcomes

The primary outcome was the incidence of PPD at 6 weeks post partum. PPD was assessed using the Edinburgh Postnatal Depression Scale (EPDS), with a score range of 0 to 30. An EPDS score greater than 10 indicated a positive PPD screening result.^19^

A secondary outcome was the incidence of PPD at 1 week post partum. EPDS scores at 1 week vs 6 weeks post partum were compared. Another secondary outcome was pain intensity, assessed using the numerical rating scale (NRS; score range: 0-10, with the highest score indicating severe pain) at postoperative day (POD) 1, 2, and 3. Pain at rest was defined as pain experienced while lying supine, while pain with movement encompassed pain experienced during activities, such as coughing or walking on level ground. Consumption of the PCIA agent and frequency of PCIA bolus administration were also recorded.

Changes in vital signs (blood pressure, heart rate, and pulse oxygen saturation) before and after the esketamine or saline infusion were collected and compared. Neonatal variables were recorded, such as sex assigned at birth; body weight; Apgar score at 1, 5, and 10 minutes after delivery; and proportion of neonatal intensive care unit transfers. In addition, any discomfort reported by each patient was documented through an open-ended inquiry conducted at the end of surgery, and the adverse events were defined based on these reported concerns. Any adverse events occurring within 6 weeks after delivery were also assessed and recorded.

### Sample Size

A power analysis was conducted based on a previous study, with the incidence of PPD at 6 weeks post partum assessed among pregnant women who had cesarean delivery.^20^ The incidence of PPD among patients without esketamine was approximately 25.7%,^20^ and an expected superiority difference of 10% (1-sided) in the incidence of PPD between patients with or without esketamine was used in the power calculation. To achieve a statistical power of 80% at a significance level of .05, we determined that 138 patients were required in each treatment group; thus, the final sample size was 154 patients in each group, considering a 10% dropout rate. The sample size was calculated using the method of superiority by a margin test for 1 proportion in PASS 15 (NCSS Statistical Software).

### Statistical Analysis

All analyses were conducted based on a modified intention-to-treat sample that included all randomized patients who received the intervention and had at least 1 EPDS score. Continuous variables were presented as either mean (SD) for normal distribution data or median (IQR) for non-normal distribution data. Categorical variables were expressed as the total number (percent frequency). To compare continuous variables, we used an unpaired, 2-tailed *t* test for normal distribution data and Mann-Whitney test for non-normal distribution data. The χ^2^ test was used for analyzing categorical variables, with differences in incidence of PPD between groups expressed as relative risk (RR) with 95% CI. Fisher exact test for categorical variables was used when the number of events was fewer than 5. Furthermore, the median difference (MD) with 95% CI in EPDS scores between groups was analyzed with the Hodges-Lehmann method.

Additionally, predefined stratified analyses (subgroups: prenatal depression, premature delivery, primipara, age ≥35 years, and type of surgery) were also performed. Regarding the screening of PPD using the EPDS, a cutoff value of 10 or higher is commonly used. However, various cutoff values have been reported in previous studies.^21,22,23^ Therefore, a sensitivity analysis was performed based on the different EPDS cutoff values for screening PPD. Statistical analyses were performed in SPSS, version 17.0 (IBM Corp). A 2-sided *P* < .05 was considered to be statistically significant.

## Results

### Baseline Characteristics and Intraoperative Variables

A total of 349 pregnant women were screened for participation, 22 of whom were excluded due to contraindications to esketamine use and 19 who refused to participate. The final sample included 308 patients who were randomly assigned to 1 of 2 groups: esketamine group (n = 154; mean [SD] patient age, 31.57 [4.26] years) and control group (n = 154; mean [SD] patient age, 32.53 [7.74] years). All patients received intervention intraoperatively and underwent at least 1 EPDS assessment after surgery. The intention-to-treat sample consisted of 154 patients in each of these 2 groups (Figure 1). Baseline characteristics of included patients are presented in Table 1, which shows no significant differences between the 2 groups. Additionally, anesthesia and surgery data are presented in eTable 1 in Supplement 2, and no significant differences in these variables were exhibited between the 2 groups. The pulse oxygen saturation of all patients remained between 99% and 100% during surgery. However, a statistical difference in median (IQR) heart rate was found before and after infusion in the esketamine vs control group (2.5 [−5 to 11] vs −1 [−10 to 5] beats per minute; *Z* = −3.26; *P* = .001). The systolic blood pressure (−1 [−7.25 to 6] vs 0 [−9 to 7.25] mm Hg; *Z* = −0.34; *P* = .73) and diastolic blood pressure (0 [−7 to 8] vs 1 [−6 to 8] mm Hg; *Z* = −0.63; *P* = .52) were comparable between the 2 groups. In addition, variables of neonates were recorded and did not differ between the 2 groups (eTable 1 in Supplement 2).

**Table 1.** Baseline Characteristics of Patients in Esketamine and Control Groups

Variable	Patients, No. (%)	
	Esketamine group (n = 154)	Control group (n = 154)
Age, mean (SD), y	31.57 (4.26)	32.53 (7.74)
BMI, mean (SD)	27.31 (3.79)	27.00 (3.22)
ASA classification		
II	140 (90.9)	143 (92.9)
III	14 (9.1)	11 (7.2)
Educational level, y		
<9	0	1 (0.7)
9-12	28 (18.8)	16 (10.4)
>12	125 (81.2)	137 (89.0)
Monthly family income, ¥		
<10 000	22 (14.3)	21 (13.6)
10 000-19 999	78 (50.7)	75 (48.7)
20 000-29 999	34 (22.1)	45 (29.2)
≥30 000	20 (13.0)	13 (8.5)
Premature delivery	11 (7.1)	11 (7.1)
Gravidity, median (IQR)	2 (1-3)	2 (1-3)
Parity, median (IQR)	0 (0-1)	0 (0-1)
Primipara	106 (68.8)	101 (65.6)
Twin pregnancy	1 (0.7)	4 (2.6)
Comorbidities		
Gestational diabetes	46 (29.9)	41 (26.6)
Anemia	6 (3.9)	8 (5.2)
Thyroid dysfunction	24 (15.6)	22 (14.3)
ICP	7 (4.5)	7 (4.5)
Arrhythmia	5 (3.2)	4 (2.6)
Attended antenatal classes	26 (16.9)	21 (13.6)
Antenatal depression	42 (27.3)	36 (23.4)
Baseline EPDS scores, median (IQR)	5 (2-10)	5 (2-9)
Baseline NRS scores, median (IQR)	0 (0-2)	0 (0-2)

Abbreviations: ASA, American Society of Anesthesiologists; BMI, body mass index (calculated as weight in kilograms divided by height in meters squared); EPDS, Edinburgh Postnatal Depression Scale (0-30, with >10 indicating a positive postpartum depression screening result); ICP, intrahepatic cholestasis of pregnancy; NRS, numerical rating scale (score range: 0-10, with the highest score indicating severe pain); ¥, yuan renminbi.

SI conversion: To convert ¥ to US$, divide by 7.33.

### Efficacy Outcomes

The incidence of positive PPD screening results at 6 weeks was significantly lower in the esketamine group compared with the control group (10.4% [16] vs 19.5% [30]; RR, 0.53; 95% CI, 0.30-0.93; *P* = .02). However, this advantage of esketamine was not observed at 1 week post partum (15.6% [24] vs 24.0% [37]; RR, 0.64; 95% CI, 0.40-1.03; *P* = .06). EPDS scores between the 2 groups demonstrated a similar pattern as the incidence of PPD, with median (IQR) scores at 6 weeks being significantly lower in the esketamine group than the control group (5 [2-7] vs 6 [3-9]; MD, −1; 95% CI, −2 to 0; *P* = .02) (Table 2).

**Table 2.** Efficacy Outcomes of Patients in Esketamine and Control Groups

Variable	Patients, No. (%)		Estimated effect (95% CI)	*P* value
	Esketamine group (n = 154)	Control group (n = 154)		
**PPD**				
1 wk	24 (15.6)	37 (24.0)	RR: 0.64 (0.40 to 1.03)	.06
6 wk	16 (10.4)	30 (19.5)	RR: 0.53 (0.30 to 0.93)	.02
**EPDS scores, median (IQR)**				
1 wk	5 (1.75 to 8)	5 (3 to 9)	MD: −1 (−2 to 0)	.08
6 wk	5 (2 to 7)	6 (3 to 9)	MD: −1 (−2 to 0)	.02
**Sensitivity analysis**				
EPDS score ≥9				
1 wk	32 (20.8)	41 (26.6)	RR: 0.78 (0.52 to 1.17)	.22
6 wk	26 (16.9)	45 (29.2)	RR: 0.57 (0.37 to 0.88)	.01
EPDS score ≥11				
1 wk	24 (15.6)	31 (20.1)	RR: 0.77 (0.47 to 1.25)	.29
6 wk	13 (8.4)	24 (15.6)	RR: 0.56 (0.29 to 1.07)	.07
EPDS score ≥12				
1 wk	15 (9.7)	23 (14.9)	RR: 0.65 (0.35 to 1.20)	.16
6 wk	8 (5.2)	18 (11.7)	RR: 0.44 (0.19 to 0.99)	.04

Abbreviations: EPDS, Edinburgh Postnatal Depression Scale (0-30, with >10 indicating a positive postpartum depression screening result); MD, median difference; PPD, postpartum depression; RR, relative risk.

Although a cutoff value of 10 or higher on the EPDS is commonly used for PPD screening, other cuffoffs were also reported. To mitigate bias, we conducted a sensitivity analysis using different cutoff values. At 6 weeks post partum, esketamine demonstrated a preventive effect against PPD with cutoff EPDS scores of 9 or higher (16.9% [26] vs 29.2% [45]; RR, 0.57; 95% CI, 0.37-0.88; *P* = .01) and 12 or higher (5.2% [8] vs 11.7% [18]; RR, 0.44; 95% CI, 0.19-0.99; *P* = .04) in the esketamine vs control group. There was also a pattern of fewer patients in the esketamine group with a positive screening for PPD when the cutoff value was set at 11 or higher; however, this advantage did not reach statistical significance when compared with the control group (8.4% [13] vs 15.6% [24]; RR, 0.56; 95% CI, 0.29-1.07; *P* = .07). Conversely, at 1 week post partum, no significant differences were observed even when the cutoff values were adjusted to 9 or higher (20.8% [32] vs 26.6% [41]; RR, 0.78; 95% CI, 0.52-1.17; *P* = .22), 11 or higher (15.6% [24] vs 20.1% [31]; RR, 0.77; 95% CI, 0.47-1.25; *P* = .29), and 12 or higher (9.7% [15] vs 14.9% [23]; RR, 0.65; 95% CI, 0.35-1.20; *P* = .16) (Table 2).

The stratified analysis of 5 factors potentially influencing the antidepressant efficacy of esketamine (prenatal depression, premature delivery, primipara, age ≥35 years, and type of surgery) did not show a significant advantage of esketamine in reducing PPD at 6 weeks post partum for any subgroup. This finding was indicated by *P* > .05 for interaction for all subgroups (eTable 2 in Supplement 2).

**Figure 2.**
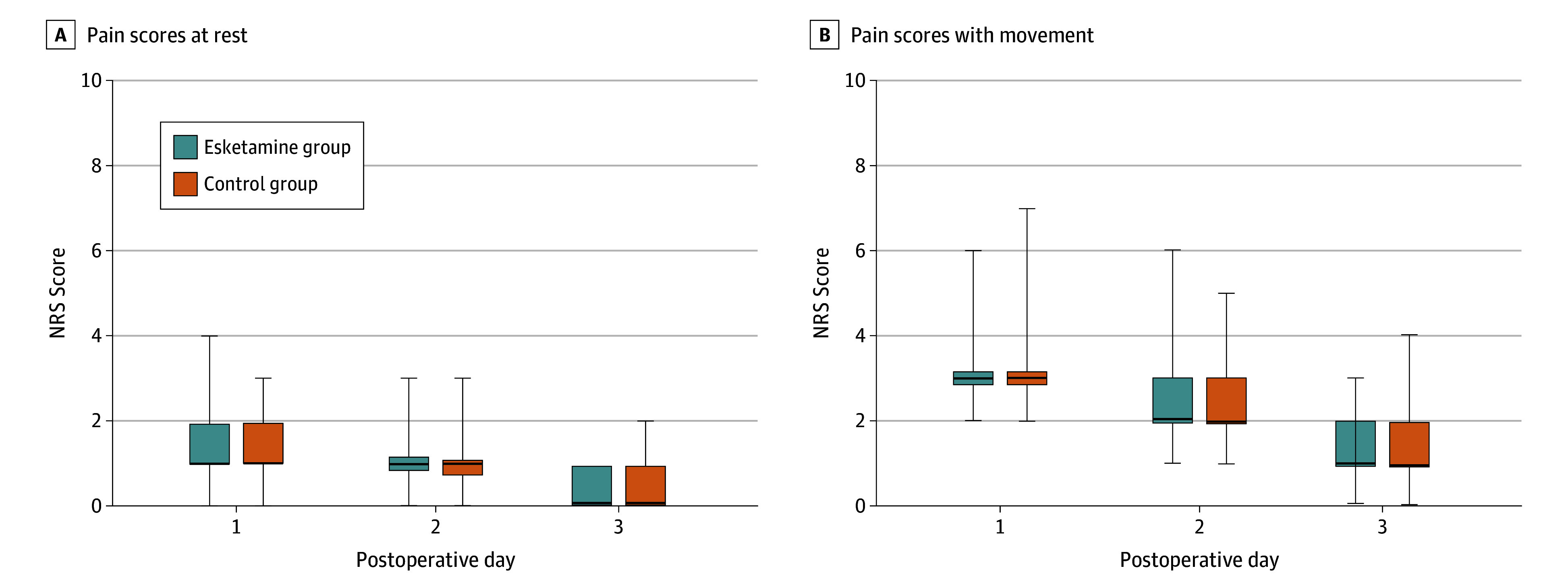
Pain Scores at Rest and With Movement Assessed by Numerical Rating Scale (NRS) at Different Postoperative Times for Esketamine and Control Groups Boxes represent lower, median, and upper quartiles; whiskers represent minimum and maximum values. At postoperative day 1, the median was 1 (the bold black line) and was covered by lower quartiles.

In addition, esketamine has been shown to possess certain analgesic effects, which were also documented in this study. Results suggested no significant difference in NRS scores for pain at rest on POD 1 (1 [1-2] vs 1 [1-2]; *Z* = −1.61; *P* = .10), POD 2 (1 [1-1] vs 1 [0.75-1]; *Z* = −0.48; *P* = .62), and POD 3 (0 [0-1] vs 0 [0-1]; *Z* = −0.59; *P* = .54) between the esketamine and control groups (Figure 2A). Similarly, no significant differences between the 2 groups were observed in NRS scores for pain with movement on POD 1 (3 [3-3] vs 3 [3-3]; *Z* = −0.26; *P* = .79), POD 2 (2 [2-3] vs 2 [2-3]; *Z* = −1.64; *P* = .10), and POD 3 (1 [1-2] vs 1 [1-2]; *Z* = −0.56; *P* = .57) (Figure 2B). The consumption of PCIA agents was also recorded. Total consumption did not differ significantly between the 2 groups (75.55 [5.81] mL vs 76.40 [6.06] mL; *t* = −1.25; *P* = .21). However, fewer instances of PCIA bolus administration were recorded in the esketamine group than the control group (58 [37.7%] vs 83 [53.9%]; χ^2^ = 8.17; *P* = .004). Additionally, the median (IQR) frequency of PCIA bolus administration was lower in the esketamine group than the control group (0 [0-2] vs 1 [0-3]; *Z* = −2.42; *P* = .01).

### Adverse Events

More adverse events were observed at the end of surgery in the esketamine group. Specifically, a higher number of patients who received esketamine infusion vs saline reported experiencing dizziness (28 [18.2%] vs 3 [1.9%]; χ^2^ = 22.41; *P* < .001), trance-like states (9 [5.8%] vs 0; *P* = .003), and dreamscape sensations (16 [10.4%] vs 0; χ^2^ = 16.87; *P* < .001). Cases of hallucination were reported in the esketamine group, although the incidence did not differ significantly compared with that in the control group (4 [2.6%] vs 0). Within 3 days post partum, adverse events were recorded in both the esketamine and control groups (23 [14.9%] vs 14 [9.1%]); however, no statistical difference was found (χ^2^ = 2.48; *P* = .11). From 3 days to 6 weeks post partum, more adverse events were reported in the esketamine group vs the control group. Nevertheless, no significant difference was observed between the groups (χ^2^ = 2.13; *P* = .14). Adverse events data are shown in Table 3.

**Table 3.** Reported Adverse Events at Different Times for Patients in the Esketamine and Control Groups

Adverse event	Patients, No. (%)		*P* value
	Esketamine group (n = 154)	Control group (n = 154)	
At end of surgery			
Dizziness	28 (18.2)	3 (1.9)	<.001
Mental trance state^a^	9 (5.8)	0	.003
Hallucination^b^	4 (2.6)	0	.12
Dreamscape^c^	16 (10.4)	0	<.001
Diplopia	1 (0.6)	0	>.99
Nausea or vomiting	7 (4.5)	9 (5.8)	.60
Total	65 (42.2)	12 (7.8)	<.001
Within 3 d post partum			
Fever	1 (0.6)	0	>.99
Dizziness	8 (5.2)	5 (3.2)	.39
Nausea or vomiting	5 (3.2)	2 (1.3)	.44
Flatulence	3 (1.9)	1 (0.6)	.62
Acroparesthesia	1 (0.6)	0	>.99
Uncontrolled pain	5 (3.2)	6 (3.9)	.75
Total	23 (14.9)	14 (9.1)	.11
From 3 d to 6 wk post partum			
Infants in hospital^d^	1 (0.6)	0	>.99
Pain complaint	6 (3.9)	7 (4.5)	.77
Anxiety	7 (4.5)	2 (1.3)	.17
Acroparesthesia	2 (1.3)	0	.49
Total	16 (10.4)	9 (5.8)	.14

^a^ Mental trance state is defined as greatly narrowed awareness and reactivity to a stimulus. A patient in this state exhibited altered consciousness and was unable to respond or follow instruction.

^b^ Hallucination is defined as experiencing something that does not exist.

^c^ Dreamscape is defined as a dreamlike scene. A patient experienced a dream during the surgery.

^d^ Not including those who were admitted immediately after giving birth.

## Discussion

This pragmatic randomized clinical trial demonstrated that among women with cesarean delivery, intraoperative esketamine infusion could reduce the incidence of positive PPD screening and EPDS scores at 6 weeks post partum in actual clinical practice. However, these benefits were not evident at 1 week post partum. Sensitivity analysis confirmed these findings. Esketamine did not demonstrate any advantage in reducing pain scores but did decrease the frequency of PCIA bolus administration. In addition, esketamine use was associated with an increase in heart rate and adverse events at the end of surgery. These adverse effects were transient and diminished quickly over time, however.

Unlike the established role of esketamine or ketamine in depression treatment, its role in PPD is still in early stages of investigation. Thus, the efficacy of esketamine in preventing PPD remains a primary concern. Previous studies have reported that patients with esketamine treatment had a lower incidence of PPD at 1 week post partum,^10,19,24^ which was inconsistent with our findings. There may be several reasonable explanations for these differences. First, different cutoff EPDS scores for PPD have been used in various studies, potentially affecting the calculation of the incidence of PPD. Second, certain studies have explored the effect of diverse modes or therapeutic doses on antidepressant properties.^25,26^ Thus, discrepancies may stem from variations in the dose, mode, and timing of esketamine administration. Third, the distinction between a pragmatic design and RCT is of utmost importance. In contrast to RCTs, pragmatic trials do not control for the confounding factors presented in the clinical setting. Consequently, the results may vary, but this underscores the distinctive value of this trial.

In addition to the overall results, a subgroup analysis revealed that esketamine was not associated with lower pain scores among the subgroups. This finding contrasts with results of the Liu et al study^27^ showing that ketamine reduced PPD incidence in high-risk cohorts but not in low-risk ones. To date, this topic remains controversial. Some studies have reported favorable outcomes of ketamine or esketamine even after excluding patients with prenatal depression.^19,28^ Furthermore, it has been established that prenatal depression was associated with a decreased probability of prevention effectiveness of ketamine or esketamine in a logistic regression model.^29^ Thus, whether ketamine or esketamine should be exclusively administered to high-risk patients warrants investigation. Due to the limitation of this single-center trial with small samples, the ongoing debate necessitates validation through large-scale, high-quality studies.

Regarding the adjunctive analgesic effect of esketamine in parturients undergoing cesarean delivery, the conclusions in RCTs were inconsistent.^8,11,19,28,30,31^ Four of 6 meta-analyses^13,14,32,33,34^ addressed the analgesic effect of esketamine or ketamine, but none reached definitive conclusions: 2 reported beneficial results,^13,32^ while the others had adverse outcomes.^14,35^ In the present trial, esketamine did not provide adjunctive analgesic effect within 3 days post partum, nor did it reduce the opioid consumption in clinical practice. This discrepancy in findings may be due to differences in pain management in actual practice wherein patients would receive various durations of analgesia based on assessment by the acute pain service team and patient preferences. Furthermore, multimodel analgesic regimens, which included adjustments to PCIA parameters, nerve block, and supplementary analgesia, varied among patients. One study indicated that intravenous esketamine could reduce pain intensity for a short period after surgery.^33^ We also evaluated the frequency of PCIA bolus administration and found that esketamine could reduce the need for bolus administration, highlighting esketamine’s potential advantage in managing breakthrough pain. Despite its lack of effect on overall pain and opioid consumption, esketamine could still offer benefits in reducing both PPD and postoperative pain among women who had cesarean delivery in clinical practice.

Regarding the adverse events associated with esketamine, especially in cardiovascular and neuropsychiatric systems, we noted a slight increase in heart rate after esketamine administration, but this change was not clinically significant. Moreover, there was no significant fluctuation in blood pressure after esketamine infusion. Conversely, more neuropsychiatric adverse events were recorded in patients who received esketamine. However, consistent with findings of a previous study,^36^ these adverse events were transient and short-lived. It has been demonstrated that the rate of administration of esketamine or ketamine is associated with its adverse events.^37^ In this trial, a slower infusion over 20 minutes resulted in a 37.7% incidence of adverse events compared with 33.5% with a 40-minute infusion^36^ and 97.7% with a 1-minute intravenous administration.^38^ This finding raises a question about the optimal administration route for esketamine to prevent PPD. Current evidence does not provide clear guidance on this issue. Recently, our team conducted a cohort study to investigate the differences in PPD incidence and adverse events between esketamine infusion and intravenous injection in women undergoing cesarean delivery in an effort to understand the optimal use of esketamine for PPD prevention (L. Ren, PhD, unpublished data, 2024).

### Limitations

This study has several limitations. First, this was a single-center pragmatic clinical trial. While the center, a prominent childbirth center in Chongqing with approximately 500 to 600 cases annually, may be representative in China, there is still a need for multicenter and large-scale trials to confirm the generalizability of this trial’s conclusions. Second, the pragmatic design of this trial was assessed using the Pragmatic Explanatory Continuum Indicator Summary-2 (PRECIS-2) tool, including 9 domains scored from 1 (very explanatory) to 5 (very pragmatic).^39^ This trial received a total PRECIS-2 score of 30, with 4 domains scored with 4, indicating some pragmatic characteristics. According to Ford and Norrie,^15^ few studies achieve a completely pragmatic design. Thus, it seemed the PRECIS-2 tool regarded this study as a pragmatic trial that partially represented clinical practice.^40^ However, extremely pragmatic trials are necessary to fully explore the efficacy of esketamine in preventing PPD in clinical practice. Third, use of professional assessment tools for PPD was restricted. EPDS was the only tool used to screen for PPD, and psychiatrists did not participate in this evaluation or provide professional diagnosis. Fourth, the follow-up time points were set at 1 week and 6 weeks post partum, However, other short-term (at 2 and 4 weeks post partum) and long-term effects of esketamine remain unknown and need further investigation.

## Conclusions

This pragmatic clinical trial found that intraoperative esketamine infusion could reduce the incidence of PPD at 6 weeks post partum among patients who underwent cesarean delivery. However, the advantage of esketamine was not evident at 1 week post partum. Esketamine seemed to have a potential adjunctive analgesic effect by reducing the frequency of PCIA bolus administration, although no significant reduction in pain scores was noted. More adverse events at the end of surgery were reported in the esketamine group than the control group. However, these adverse effects were generally tolerable and diminished quickly over time. The efficacy and safety of esketamine in preventing PPD warrant further investigation in actual clinical practice.

## Notes

Supplement 1.
Trial Protocol

Supplement 2. **eTable 1.** Intraoperative Variables of the Subjects and Neonatal Outcomes in Esketamine and Control Group **eTable 2.** Effect of Esketamine vs Control on PPD in Stratified Analysis

Supplement 3.
Data Sharing Statement
