# Supplementary material for: Intraoperative Esketamine and Postpartum Depression Among Women With Cesarean Delivery: A Randomized Clinical Trial
Source: JAMA Netw Open. 2025 Feb 13;8(2):e2459331. doi: 10.1001/jamanetworkopen.2024.59331 (PMC11826358; doi:10.1001/jamanetworkopen.2024.59331)
Supplement: Supplement 1. — Trial Protocol [file jamanetwopen-e2459331-s001.pdf]

Intraoperative Esketamine and Postpartum Depression Among Women With Cesarean Delivery: A Pragmatic Randomized Clinical Trial

Study protocol

Principal investigator: Ping Li, MD, Fangliang Peng, MS, Gangming Wu, MS

Organizer institutes: Department of Anesthesiology, The First Affiliated Hospital of Chongqing Medical University

Research Institutions: The First Affiliated Hospital of Chongqing Medical University

Content

1. Background 3

2. Objectives 4

3. Study design 4

3.1 Type of study 4

3.2 Sample size 5

4. Participants recruitment 5

4.1 Inclusion criteria 5

4.2 Exclusion criteria 6

5. Randomization and blinding 6

6. Groups and intervention 6

7. Study procedures 6

8. Primary outcomes 7

9. Secondary outcomes 7

10. Quality control 8

11. Statical analysis 8

1. Background

Postpartum depression is a common mental disorder during puerperium. A large number of postpartum depression patients in China have not been detected. Most women have negative emotions such as depression, anxiety, fear, and pessimism. They have disharmonious relationships with their husbands and feel desperate about life. In severe cases, they may even have suicidal or infanticidal tendencies, which have serious adverse effects on the physical and mental health of women and can lead to decreased cognitive abilities of infants and an increased risk of adverse cognitive outcomes for children. This seriously threatens the health of mothers and infants and increases social health costs. Therefore, it is very important to discover, diagnose, prevent, and treat postpartum depression early.

Currently, the primary approach for treating postpartum depression is pharmacotherapy. However, most medications have a slow onset, require long-term treatment, exhibit a low clinical remission rate, are prone to chronicization, and have many residual symptoms. Addressing the shortcomings of existing antidepressants, the U.S. Food and Drug Administration (FDA) approved the first antidepressant with a novel mechanism of action, esketamine, on March 4, 2019, for the treatment of adult patients with TRD (treatment-resistant depression). Esketamine exhibits a stronger specificity for NMDA receptors, which may result in a stronger antidepressant effect. Esketamine is the S enantiomer of racemic ketamine and is primarily metabolized by CYP450. Most of its metabolites (≥78%) are excreted through urine, while a small portion (≤2%) is excreted through feces, indicating minimal impact on lactation. Numerous preclinical studies have been conducted on ketamine and its enantiomer S-ketamine (esketamine), all of which suggest that they can effectively improve depressive symptoms in mice. Multiple high-quality randomized

clinical trials (RCTs) have investigated the effect of esketamine on the PPD in women with cesarean delivery, however, the conclusions were inconsistent. Furthermore, a growing number of researchers are attempting to use meta-analysis to conclude the roles of ketamine/esketamine in PPD, and positive conclusions were exhibited in recent published meta-analysis. As a result, RCTs have become the gold standard tool for proving the efficacy and safety of treatment. However, in actual clinical practice, patients exhibit different variables, and not all patients are behaved as the representative subjects designed in RCTs. Additionally, the practice defined by RCTs significantly differs from the real-world clinical practice. All these factors may mask the effectiveness of using esketamine in PPD in actual clinical setting.

Thus, we want to conducted a pragmatic clinical trial (PCT) to explore the real clinical efficacy of intraoperative esketamine usage for preventing PPD in women undergoing cesarean section with intraspinal anesthesia. We hypothesized that patients with esketamine usage exhibited lower incidence of PPD, as measured by Edinburgh Postnatal Depression scale, compared to those without esketamine usage.

2. Objectives

To explore the real clinical efficacy of intraoperative esketamine usage for preventing PPD in women undergoing cesarean section with intraspinal anesthesia.

3. Study design

3.1 Type of study

This study is a single-centered, double-blinded, placebo-controlled, pragmatic, clinical trial. The flow chart of study is shown as follow:

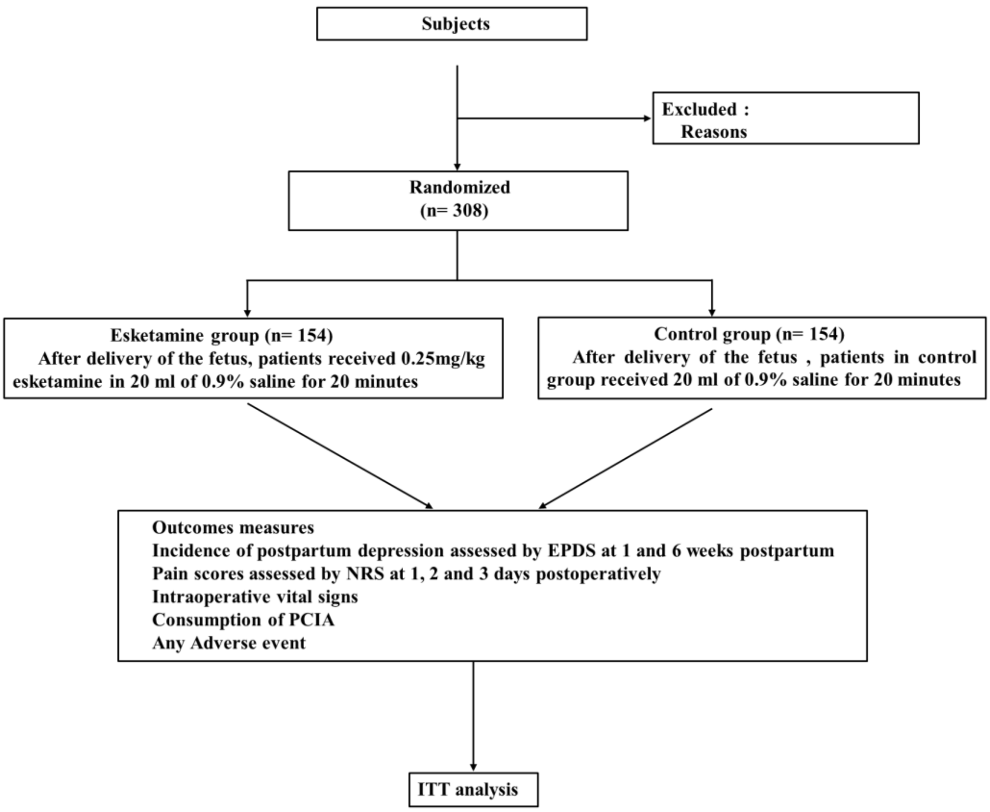

3.2 Sample size

A power analysis was performed based on our previous study, with the incidence of PPD at 6 weeks postpartum among pregnant women undergoing cesarean section. The incidence of PPD among subjects without esketamine was approximately 25.7%, and an expected superiority difference of 10% (one-side) in incidence of PPD between subjects with or without esketamine was used in the power calculation. To achieve a statistical power of 80% at a significant level of 0.05, it was determined that 138 patients were required in each group, and the final sample size was 154 patients considering 10% drop out. The sample size was calculated using the method of superiority by a margin test for one proportion in PASS 15.

4. Participants recruitment

4.1 Inclusion criteria

Pregnant women who were admitted for cesarean deliveries with intraspinal anesthesia were eligible.

4.2 Exclusion criteria

(1) severe cardiovascular or cerebrovascular diseases, severe gestational hypertension, preeclampsia or eclampsia;

(2) intragastric, intraocular or intracranial hypertension;

(3) uncontrolled hyperthyroidism;

(4) a history of drug abuse;

(5) intellectual dysfunction。

5. Randomization and blinding

All eligible subjects were assigned randomly to esketamine group or control group in a 1:1 ratio using a computer-generated random number sequence, and the group information was sealed in opaque envelopes. The assistant who helps to prepare the agents and outcomes assessors were blinded to the group assessments. And the anesthesiologists and healthcare team members involved in conducting cesarean delivery were not involved in the follow-up surveys.

6. Groups and intervention

After delivery of the fetus, patients received the different intervention. Esketamine group: patients received 0.25mg/kg esketamine in 20 ml of 0.9% saline for 20 minutes; Control group: patients received 20 ml of 0.9% saline for the same duration.

7. Study procedures

All the patients receive routine perioperative maternal practice. Before surgery and

anesthesia, the consent is obtained. After operation room admission, the patient is placed in a lateral position and intraspinal anesthesia is performed by an anesthesiologist. A target sensory block level is set as from T6 to T4. Vasoactive agents are used when necessary. And routine cesarean delivery is administrated with the clinical experience of obstetricians. Forceps, B-lynch suture and uterine balloon tamping are allowed to be used when necessary. After delivery of the fetus, the intervention is administrated according the groups assignment. A patient-controlled intravenous analgesia (PCIA) is provided for each patient at the end of surgery. And after surgery, the patients received routine clinical practice by the obstetricians

8. Primary outcomes

The primary outcome is the incidence of PPD at 6 weeks postpartum. In this study, PPD was screened by Ediburgh Postnatal Depression Scale (EPDS, scores ranging from 0 to 30) scores of more than 10

9. Secondary outcomes

The secondary outcome is the incidence of PPD at 1 weeks postpartum. EPDS scores at 1 and 6 weeks postpartum were also assessed in this study. In addition, other predefined secondary outcome includes pain intensity assessed by numerical rating scale (NRS) at 1, 2, 3 days postpartum. The pain at rest is defined as patient felt painful while lying in a supine position on the bed, while the pain with movement is considered as the patient felt painful when cough or walking on the level ground. And the PCIA agent consumption and the frequency of PCIA bolos are also recorded. The intraoperative vital signs and any adverse events are recorded. The information about

the infants is also required to be recorded.

10. Quality control

All the data will be kept for at least 2 years. If any other researchers have any diabetes about this study, they can contact the corresponding authors for access to the original data. All the data related to patients will be archived and safeguarded. The protocol will be reviewed and revised by statistical experts (Pro Yaoyue Hu, and Yi Tao). And the visitors for the follow-up will be trained. The Ethics consent and clinical registration will be done before the trial administration.

11. Statical analysis

All analyses were conducted based on modified intention-to-treat (ITT) sample that comprised all randomized subjects received intervention and had at least one EPDS scores. Data were presented as either the mean ± standard deviation (for normal distribution data) or median (interquartile range) (for non-normal distribution data) for continuous variables. Categorical variables were expressed as the total number (percent frequency). To compare continuous variables, the t-test was used for normal distributed data, while rank-sum test was used for non-normal distributed data. The chi-square test was employed for analyzing categorical variables, with differences of incidence of PPD between groups expressed as relative risk (RR) and 95% confidence interval (CI). Furthermore, the median difference and 95% CI of EPDS scores between groups was analyzed with the method of Hodges-Lehmann. And a subgroup analyses and sensitivity analyses will be conducted. Statistical analyses were performed in SPSS (version 17.0; SPSS Inc., Chicago, III). $P < 0.05$ was considered statistically

significant.
