# Supplementary material for: Intraoperative Esketamine and Postpartum Depression Among Women With Cesarean Delivery: A Randomized Clinical Trial
Source: JAMA Netw Open. 2025 Feb 13;8(2):e2459331. doi: 10.1001/jamanetworkopen.2024.59331 (PMC11826358; doi:10.1001/jamanetworkopen.2024.59331)
Supplement: Supplement 2. — eTable 1. Intraoperative Variables of the Subjects and Neonatal Outcomes in Esketamine and Control Group eTable 2. Effect of Esketamine vs Control on PPD in Stratified Analysis [file jamanetwopen-e2459331-s002.pdf]

Supplemental Online Content

Ren L, Zhang T, Zou B, et al. Intraoperative esketamine and postpartum depression among women with cesarean delivery: a pragmatic randomized clinical trial. *JAMA Netw Open*. 2025;8(2):e2459331. doi:10.1001/jamanetworkopen.2024.59331

eTable 1. Intraoperative Variables of the Subjects and Neonatal Outcomes in Esketamine and Control Group

eTable 2. Effect of Esketamine vs Control on PPD in Stratified Analysis

This supplemental material has been provided by the authors to give readers additional information about their work.

© 2025 Ren L et al. *JAMA Network Open*

eTable 1. Intraoperative variables of the subjects and neonatal outcomes in esketamine and control group

**Variables**	**Esketamine group (n=154)**	**Control group (n=154)**	***P* values**
Type of surgery			0.59
elective surgery	115 (74.67%)	119 (77.27%)	
emergency surgery	39 (25.33%)	35 (22.73%)	
Mode of surgery			0.14
Routine	134 (87.0%)	119 (77.27%)	
With B-lynch suture	8 (5.19%)	17 (11.03%)	
With forceps help	11 (7.14%)	16 (10.38%)	
With uterine balloon	1 (0.67%)	2 (1.32%)	
Duration of surgery	42.17±10.20	43.46±12.37	0.32
Type of anesthesia			0.16
Epidural anesthesia	12 (7.79%)	16 (10.38%)	
Spinal anesthesia	18 (11.7%)	9 (5.86%)	
Combined spinal-epidural anesthesia	124 (80.51%)	129 (83.76%)	
Duration of anesthesia	82.95±14.60	84.55±15.25	0.34
Nerve block at the end of surgery	17 (11.03%)	21 (13.63%)	0.48
Difference between before and after agent infusion			
SBP (mmHg)	-1 (-7.25~6)	0 (-9~7.25)	0.73
DBP (mmHg)	0 (-7~8)	1 (-6~8)	0.52
HR (beat/min)	2.5 (-5~11)	-1 (-10~5)	0.001
Neonatal outcomes			
Sex (M/F)	86/68	91/63	0.56
Body weight (g)	3262.66±405.48	3181.38±452.28	0.09
Apgar scores			
1 min	10 (9-10)	10 (9-10)	0.64
5 min	10 (10-10)	10 (10-10)	1.00
10 min	10 (10-10)	10 (10-10)	0.31
Transferred to NICU	9 (7.18%)	8 (7.28%)	0.80

SBP: diastolic blood pressure; DBP: systolic blood pressure; HR: heart rate; NICU: neonatal intensive care unit

eTable 2. Effect of esketamine vs control on PPD in stratified analysis

Subgroup	Esketamine group	Control group	RR (95%CI)	*P* value for interaction
All patients	16/154	30/154	0.53 (0.30-0.93)	/
Prenatal depression				1.00
Yes	2/42	17/36	0.10 (0.02~0.40)	
no	14/112	13/118	1.13 (0.55~2.30)	
Premature delivery				0.99
Yes	0/11	5/11	0.09 (0.006~1.46)	
No	16 /143	25/143	0.64 (0.35~1.14)	
Primipara				0.42
Yes	10/106	23/101	0.41 (0.20~0.82)	
No	6/48	7/53	0.94 (0.34~2.60)	
Age				0.23
< 35	11/118	27/116	0.40 (0.20~0.76)	
⩾35	5/36	3/38	1.75 (0.45~6.83)	
Surgery				0.93
Elective	12/115	22/119	0.56 (0.29~1.08)	
Emergency	4/39	8/35	0.44 (0.14-1.36)	

© 2025 Ren L et al. *JAMA Network Open*
