# Supplementary material for: Intraoperative Esketamine and Postpartum Depression Among Women With Cesarean Delivery: A Randomized Clinical Trial
Source: JAMA Netw Open. 2025 Feb 13;8(2):e2459331. doi: 10.1001/jamanetworkopen.2024.59331 (PMC11826358; doi:10.1001/jamanetworkopen.2024.59331)
Supplement: Supplement 3. — Data Sharing Statement [file jamanetwopen-e2459331-s003.pdf]

Data Sharing Statement

Ren. Intraoperative Esketamine and Postpartum Depression Among Women With Cesarean Delivery. *JAMA Netw Open*. Published February 13, 2025.
doi:10.1001/jamanetworkopen.2024.59331

Data
Additional Information: Chinese Clinical Trial Registry Identifier: ChiCTR2200065494
Data available: Yes
Data types: Deidentified participant data
How to access data: to the email: wugangming@126.com
When available: With publication

Supporting Documents
Document types: Statistical/analytic code
How to access documents: to the email: wugangming@126.com
When available: With publication

Additional Information
Who can access the data: researchers whose proposed use of the data has been approved
Types of analyses: non-commercial purposes
Mechanisms of data availability: with a signed data access agreement
